# Procyanidins from Cranberry Press Residues—Extraction Optimization, Purification and Characterization

**DOI:** 10.3390/plants11243517

**Published:** 2022-12-14

**Authors:** Linards Klavins, Ingus Perkons, Marcis Mezulis, Arturs Viksna, Maris Klavins

**Affiliations:** 1 Department of Environmental Science, University of Latvia, LV-1004 Riga, Latvia; 2 Institute of Food Safety, Animal Health and Environment “BIOR”, LV-1076 Riga, Latvia; 3 Faculty of Chemistry, University of Latvia, LV-1004 Riga, Latvia

**Keywords:** American cranberry, *Vaccinium macrocarpon*, procyanidins, optimization, response surface methodology, antioxidants, press residues, valorization

## Abstract

Procyanidins are a polyphenolic group that can be found in a variety of foods such as chocolate, tea, cranberries and others. Type A procyanidins can be found in a handful of sources and one of the richest sources are American cranberries. These compounds possess antioxidative, anticancer and anti-inflammatory activities and are most widely used as prevention for urinary tract infections. Cranberries are utilized for jam and juice production, and the latter produces industrial food waste press residues. Press residues contain free and bound procyanidins which can be extracted for use as nutraceuticals. In this study, the extraction of cranberry press residues has been optimized using RSM and the resulting extracts have been purified and fractionated. The obtained procyanidin fractions have been investigated for their antioxidative potential and analyzed using LC-ESI-FTICR-HRMS to determine individual procyanidins. The optimization showed that the optimal extraction can be conducted using acetone in a concentration of 53% without the addition of an acidifying agent. Strong correlation was observed for procyanidin contents and their antioxidative activity using DPPH, ABTS and FRAP methods. The purified fractions contained 78 individual (65 Type A) procyanidins with the degree of polymerization of up to 9.

## 1. Introduction

Procyanidins (PCs) are polyphenolics composed of flavan-3-ol or flavan-3,4-diol units and can be described as condensed tannins. Basic monomeric structures forming PC are (+) catechin and (−) epicatechin, but PCs can be found as monomers, oligomers with a polymerization degree between 2 and 10 and polymers with a polymerization degree higher than 10 [1]. PCs can be found in leaves, tree bark and plants, different fruits, vegetables, grains and other kinds of plant material, differing in composition and polymerization degree [2,3,4,5]. PCs in plants can be found as free substances or bound to cell membranes or other biopolymers such as proteins, cellulose and hemicellulose. Free PCs have lower molecular weight than bound ones. In plants, PCs play a role in defense against oxidative stress, UV radiation and herbivores [6]. PCs are an important component of our diets primarily as strong antioxidants; they can prevent lipid oxidation; however, in many studies, their anti-inflammatory, anticancer, antimicrobial and other activities have been demonstrated [7,8,9]. Biological and pharmacological activities of PC determine their use in preventing urinary tract infections (UTIs) [7], in regulation of blood pressure [10] and supporting cardiovascular health [11], as well as their use as sunscreens and in cosmetic applications [12]. American cranberries (*Vaccinium macrocarpon* L.) have been shown to contain PCs, specifically, those of the A-type: a type of PC which contains an additional ether interflavan bond between C2 and C7. Cranberries are one of the few sources of A-type PCs, which are the molecules that are responsible for the protection against UTIs. While many products contain procyanidins of the B-type, the specific anti-adhesion activity against uropathogenic *E. coli* are associated with the PCs of A-type and, in fact, the B-type procyanidins show very little activity [13,14].

Cranberry press residue is a by-product of the juice industry. It consists of seeds skins and residual flesh. This material contains free and bound polyphenolics, including procyanidins. To effectively retrieve the bioactive procyanidins from press residues, extraction techniques and conditions must be examined in the terms of utilizing solutions proposed by bioeconomy based biorefining. Considering diverse application possibilities, many extraction options are already elaborated, differing in their approaches; some support the extraction of free PCs, some of bound, different types of used solvent systems are employed, as well as fractionation approaches to obtained PC mixtures [15,16]. Another challenge in the production of PCs is related to the need to prevent their oxidation during the extraction process, supporting production of as intact PC molecules as possible. Traditionally, extraction with 70% acetone and addition of acetic acid or other acidifying agent has been used [17]; however, the development of extraction systems to obtain higher yields of PCs is in progress [18]. Recently, intensive extraction methods like ultrasound assisted, microwave and high voltage electric field extraction have been tested [19,20]. Moreover, subcritical and supercritical fluid extraction [21] have been applied on different PC sources as well as deep eutectic solvents and ionic liquids [18]. Isolation of bound PCs requiring destruction of hydrogen, hydrophobic, ionic and sometimes even covalent bonds between PCs and the structural polysaccharides is another challenge that procyanidin research faces. Hydrolytic treatment (acid or enzymatic hydrolysis) could provide the required results for the extraction of bound procyanidins [22]. Despite the bound PCs being the larger part of total procyanidins in different types of biomass, the free, lower DP and molecular weight PCs have higher bioavailability and activity, and are therefore of interest in most studies [17].

The aim of this study was to compare different extraction solvents used for the extraction of procyanidins, as well as to identify and optimize (RSM) the extraction solvent variables most suitable for the extraction of cranberry press residues. The optimal extract was purified, and its qualitative composition was demonstrated using LC-FTICR-HRMS. Additionally, the purified fractions were analyzed for their antioxidative effects and compared with the procyanidin contents.

## 2. Results

### 2.1. Screening of Extraction Solvent Variables

Extraction of procyanidins has been conducted using a variety of solvents and solvent additives depending on the material to be extracted, the types of procyanidins or the degree of procyanidin polymerization in the specific biomass. Cranberry procyanidins have been extracted using mainly acetone at different concentrations occasionally adding different acids to lower the pH of the extraction medium [17]. The variety of the used solvents suggest the need for extraction solvent standardization for cranberries and, more specifically, cranberry press residues, as the latter is the type of biomass with the greatest potential for the extraction of the valuable procyanidins used in UTI prevention and treatment.

**Figure 1:**
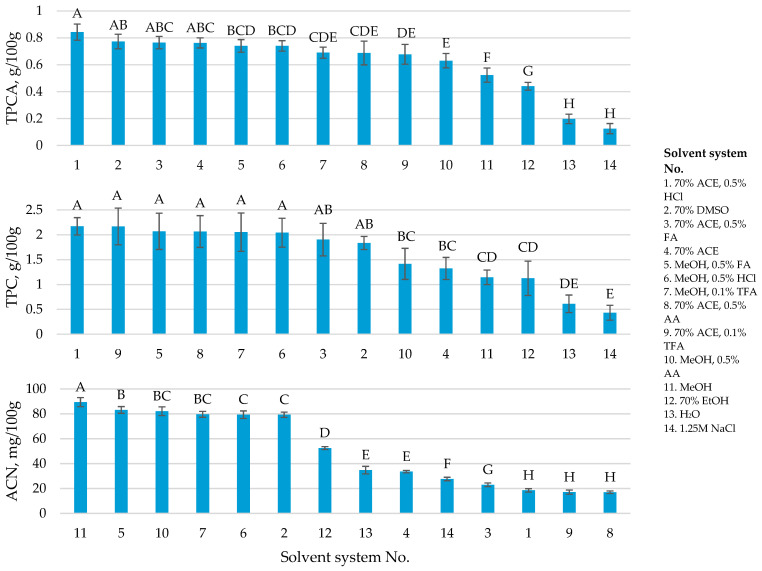
Screening of solvent compositions used for the extraction of procyanidins (TPCA) from American cranberry press residues. Additional measurements of total polyphenolics (TPC) and anthocyanins (ACN) have been conducted. The extractions were performed in triplicate. The means have been compared using ANOVA with post-hoc Tukey’s LSD test. Connecting letters above bars represent statistically different results (*n =* 3).

A literature survey was conducted to poll the solvents used for procyanidin extraction in other studies and, in order to be able to compare the extraction solvent effectiveness, the same conditions were then used on dried cranberry press residues for the extraction. The solvents for the procyanidin extraction were acetone (ACE), methanol (MeOH), ethanol (EtOH), dimethyl sulfoxide (DMSO), water and 1.25 M NaCl solution with the addition of an acidifying agent, namely acetic acid (AA), formic acid (FA), hydrochloric acid (HCl) or trifluoroacetic acid (TFA). To monitor the extracted groups of polyphenolics, the total anthocyanin contents in the extracts were also determined. Extraction solvent screening revealed that solvents containing methanol and an acidifying agent were the best for the extraction of anthocyanins and total polyphenolics. Highest extraction yields of procyanidins (up to 0.84 g/100 g) were obtained while using 70% acetone with 0.5% HCl (Figure 1). Acetone was the most effective solvent for the extraction of procyanidins in all the used combinations (with different added acids). In certain cases, where pure water or 1.25 M NaCl solution was used for the extraction of procyanidins, the extraction yields were significantly lower (up to five times lower than the most effective solvent system used in screening). Based on the solvent screening, acetone was chosen as the solvent and formic acid (FA), acetic acid (AA) and hydrochloric acid (HCl) were chosen as the acidifying agents for optimization by response surface methodology (RSM), undertaken to find the true optimum of these solvents for procyanidin extraction (Figure 1).

### 2.2. Optimisation of Extraction Solvent Using RSM

The combined model effects of the previously chosen extraction variables were examined using response surface methodology (RSM). A central composite design (CCD) of 56 runs with 6 central points was performed at a randomized order. The summary of the ANOVA analysis of the used design can be found in Materials and Methods Section 4.7. The obtained *F* an *p* values show model significance; therefore, the model can be used to predict total procyanidin contents under any combinations of the optimized variables indicating adequate accuracy and general availability of the designed polynomial models (Section 4.7).

Investigated variables *X*_1_, *X*_2_ and their interactions (Section 4.7) were tested for their significance. In all the prepared models, acetone (*X*_1_) showed a significant effect on the measured response, while the added acid (*X*_2_) showed an insignificant effect on the response. The interaction between the acid contents (for all the studied acids) and the acetone contents were insignificant in all three models (Table 1).

**Figure 2:**
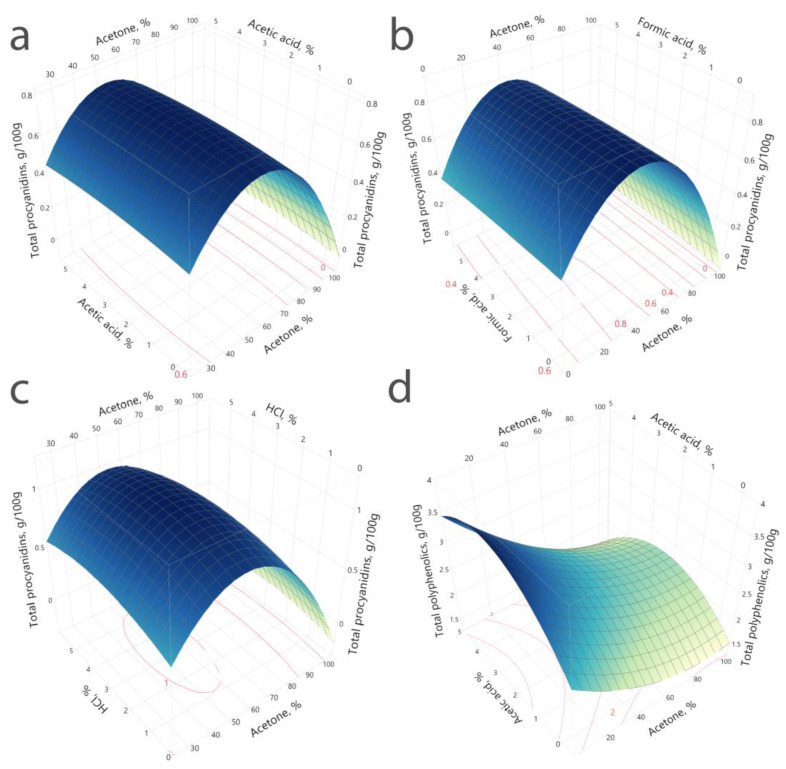
Response surface graphs showing the effect of acetone (*X*_1_) and added acid (*X*_2_) concentration on the measured response (total procyanidins): (**a**) shows the effects of adding acetic acid; (**b**) formic acid; (**c**) hydrochloric acid; (**d**) shows the effects of adding acetic acid and acetone concentration on the yield of total polyphenolics.

Obtained response surfaces visually show that the effects of the added acid, whether it is acetic, formic or hydrochloric acid, are negligible (Figure 2). Interestingly, the type of used acid can increase the amount of extracted polyphenolics; however, this addition is not concentration based. In the case of the added organic acids acetic and formic acid, the maximum extracted (predicted) total procyanidin yields is up to 0.86 g/100 g. Hydrochloric acid, when used with acetone to extract procyanidins, gives the maximum yield of 0.94 g/100 g (Figure 2).

Response surfaces and the calculated prediction formulas were used to maximize the desirability of the used variables. Since it was shown that the interaction between *X*_1_*X*_2_ and *X*_2_ showed insignificant effects on the response (total procyanidins), the values along the tested acid concentration range show statistically similar results. While using the desirability profiler, the highest theoretical extraction yields were achieved by using 2.5% hydrochloric acid and 2.3% acetic acid with 53.7% and 53.3%, respectively (Table 2). However, since the acid addition is not a significant variable in the procyanidin extraction, acid could be avoided since extraction yield would not increase. The optimal acetone concentration for the extraction of procyanidins was 52.8–53.7% (Table 2). The similar acetone concentrations for optimal extraction show that the extraction solvent should be close to these values, since all three separate models show the same values. The addition of acid should be avoided since no positive effects on the extraction yields of procyanidins could be seen.

As an example of the importance of acid addition to the extraction medium, Figure 2d shows the effects of acetic acid on the polyphenolic yields. As opposed to the procyanidin extraction for the polyphenolics, the addition of an acidifying agent shows a concrete increase of the extraction yield. The effects test shows that, in the polyphenolic’s extraction, both variables *X*_1_*X*_2_ have a significant effect on the response (*p* ≤ 0.001). Acidification supports the protonation of phenolic hydroxyl groups and thus the stabilization of procyanidins in respect to oxidation; however, changes of concentration for the acidifying agent, as well as its acidity (pH), do not have major impact on the extraction yield; release of membrane- and otherwise-bound procyanidins with studied acid concentrations is not supported, as is in the case of polyphenolic extraction.

### 2.3. Purification and Fractionation of Procyanidins

**Figure 3:**
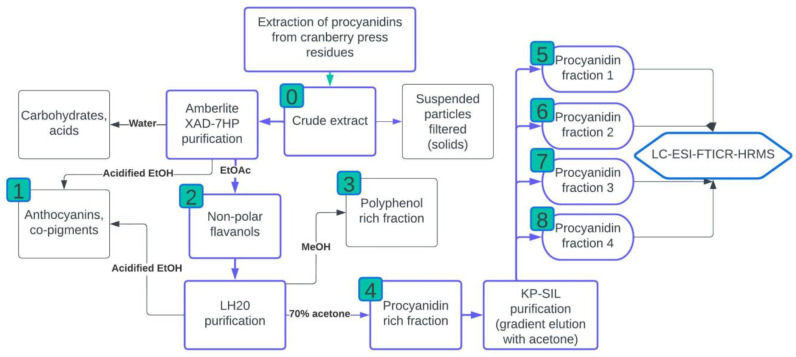
The purification of the prepared optimal procyanidin extract from cranberry press residues. Solvents indicated on the arrows are the solvents used for the elution of the specific fraction. The number indicated in green boxes are the fraction (purification steps) numbers represented in Table 3 with representative quantitative data.

Assessment of procyanidin content was conducted on an extract prepared using the optimized extraction solvent for the extraction of cranberry press residues. Since no differences were observed when any of the RSM optimized acid additions were used, only acetone was used as the extraction solvent in the concentration of 54%. The aim of the extract purification and fractionation was to obtain a strictly procyanidin containing fraction to be analyzed using LC-HRMS. Since the obtained press residue extracts are a complex mixture, several purification and fractionation steps were employed, which allowed the isolation of procyanidins of different degrees of polymerization (Figure 3).

The crude extract was firstly purified using XAD7-HP resin to remove the carbohydrates and organic acids that are present in large quantities in press residues and can be up to 40% of the total amount of the crude extract (Figure 3). Another part of the extract was the anthocyanins which were determined to be approximately 0.3% of the press residues (Table 3) and were eluted from the resin column using acidified ethanol (it was shown later that part of the procyanidins of interest were lost during this washing step; however, it was associated with the resin efficiency rather than the elution step itself, since the resin particles are comparably large and therefore the binding capacity is lower than for smaller particle resins). At purification step 2, which was elution from XAD resin using ethyl acetate (EtOAc), the extract fraction was determined to be rich in procyanidins and total polyphenolics (Table 3). Since LH20 has been widely used in purification of procyanidins, the LH20 resin was used to separate a polyphenolic and procyanidin rich fractions using methanol and acetone as eluents, respectively. Purification step 3 showed high procyanidin concentrations; however, it was confirmed by high-performance liquid chromatography with fluorescent detection (HPLC-FL) analysis that this fraction did not contain polymeric procyanidins. On the other hand, the extract fraction obtained in purification step 4 showed high procyanidin concentrations and we confirmed using HPLC-FL that it contained procyanidins with the degree of polymerization (DP) 1–10. The obtained procyanidin rich fraction, after the LH20 purification, was further fractionated using silicagel resin into four fractions that were visibly separated in UV light on the KP-SIL column. A distinct band that was eluted with 50% ethanol was determined to be a variety of hydrocinnamic and coumaric acids, which were discarded as waste (Figure 3). The four obtained fractions (purification steps 5–8) were expected to have procyanidins of different DPs; however, it was confirmed by HPLC-FL analysis, that the fractions contained the same types of procyanidins but in various concentrations (Table 3). The obtained fractions were pooled and further analyzed using liquid chromatography electrospray ionization Fourier transform ion cyclotron resonance high resolution mass spectrometry (LC-ESI-FTICR-HRMS).

### 2.4. Antioxidative Activity of Extract Fractions

Berry polyphenolics have previously been shown to have good antioxidative capabilities. Procyanidins, on the other hand, are associated with the protective effects against UTI bacteria; however, they are also shown to have radical scavenging properties [23]. To confirm this hypothesis, obtained extracts from the RSM optimization and fractionation process were analyzed for their antioxidative activity using DPPH, ABTS and FRAP methods.

**Figure 4:**
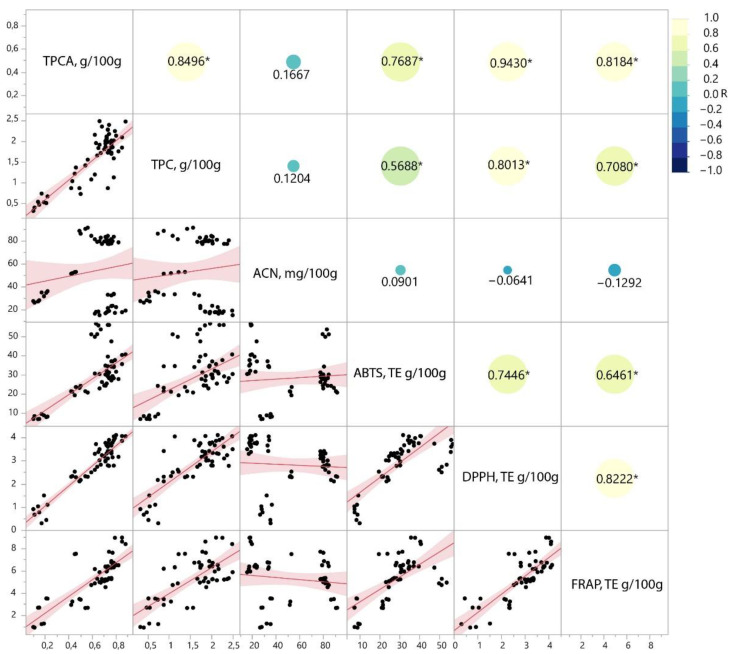
Multivariate correlation analysis of total group parameters (TPCA, ACN, TPC) and the measured antioxidative activity (DPPH, ABTS, FRAP) of extract fractions. The asterisk (*) next to the correlation coefficient R represents a statistically significant correlation (pairwise correlations, α = 0.05, *n* = 76 for each parameter).

Multivariate correlation analysis was used to see possible relations between the measured group parameters (total procyanidins (TPCA), total polyphenolics (TPC), total anthocyanidins (ACN)) and antioxidative activity measured by DPPH, ABTS and FRAP). The obtained correlation analysis showed that anthocyanins in cranberry press residues do not contribute to the measured antioxidative activity. Total polyphenolic contents, as expected, showed high correlation with the antioxidative effects for all three of the used methods; all of the correlations showed statistical significance (Figure 4). Since anthocyanins showed negligible correlation to the antioxidative potential and total polyphenolics showed significant correlation, it can be concluded that another group of polyphenolics is responsible for the antioxidative effects of polyphenolics. Measurements of total procyanidins (TPCA) showed significant correlation with the TPC contents, indicating that procyanidins were also measured as part of the total polyphenolics and that the TPC antioxidative effects could be attributed to the procyanidin contents of the extracts/fractions. Total procyanidin contents showed statistically significant correlations with ABTS, DPPH and FRAP antioxidative activity measurements, which were higher than those of the TPC measurements, indicating that cranberry press residue procyanidins are the main antioxidants in the extracts and/or their fractions (Figure 4).

### 2.5. Qualitative Characterization of Procyanidins by LC-ESI-FTICR-MS

In this study, the qualitative analysis of the procyanidin-rich fraction of *Vaccinium macrocarpon* was carried out by LC-HRMS in negative ESI mode. To simplify the processing of full-MS data, a peak-picking threshold was set to 2.5% in relation to the most intense signal detected in base peak chromatograms. In accordance with Lin et al. (2014), molecular formulas of PCs follow a well-established set of rules and can be calculated from the following features: degree of polymerization (DP), flavan-3-ol composition in the oligomer, the number of galloyls and the number of A-type linkages in the structure [24]. The aforementioned rules were used to elucidate molecular formula and qualitatively characterize the underlying PC compound. Special attention was paid to distinguish singly charged [M-H]^−^ species from double charged species [M-2H]^2−^, since the latter can interfere with the former (and vice versa). For example, [M-H]^−^ species of an A-type dimer of (epi-)catechin (C_30_H_24_O_12_) and [M-2H]^2−^ species of an A-type tetramer of (epi-)catechin (C_60_H_48_O_24_) both produce the most abundant signal at m/z 575.1195. The only difference is the observed isotopic pattern: the second most abundant ion for singly charged species of the dimer and double charged species of the tetramer is at m/z 576.1229 and m/z 575.6212, respectively. Thus, the qualitive assessment of full-MS data cannot rely on the first isotopologues alone.

**Figure 5:**
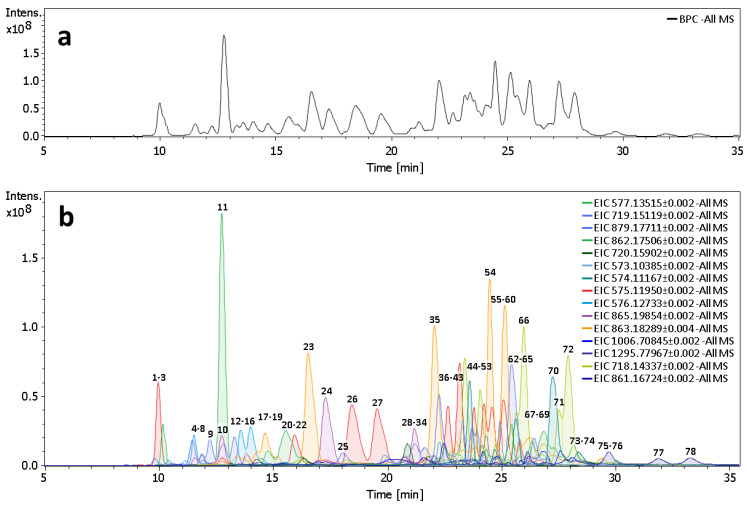
(**a**) Base peak chromatogram and (**b**) extracted ion chromatograms of procyanidin oligomers found in *Vaccinium macrocarpon* purified press residue extract by LC-HRMS. The numbers above peaks represent the identified procyanidins in Table 4.

As depicted in Figure 5, 78 peaks were detected. Molecular formula assignment revealed the presence of eighteen isomeric groups, all belonging to PC family (data summarized in Table 4). The most frequently detected PCs were pentamers (*n* = 15) and tetramers (*n* = 11) with one A-type linkage, followed by hexamers (*n* = 8) and pentamers (*n* = 7) with two A-type linkages. Nevertheless, the most intense signal was found for compound 11, which was putatively identified as B-type (epi)catechin dimer. In fact, 76 out of 78 compounds contained (epi)catechin as the sole monomeric unit (Table 4). Only compounds 1 and 7 (trimers with one A-type linkage) contained (epi)gallocatechin as one of the monomers. Singly charged species of (epi)catechin were also detected but did not exceed the peak-picking threshold. No traces of (epi)gallocatechin, (epi)afzelechin or PCs containing (epi)afzelechin were found.

To further verify the tentative identities of detected PCs, ddMS2 experiments were carried out at three collision energy levels. Comprehensive fragmentation spectra were obtained for 75 out of 78 compounds, confirming the presence of characteristic fragment ions that can be attributed to PCs. For example, ten most frequently detected fragments across ddMS2 dataset (Figure S1 in the Supplementary Materials) are related to the proposed PC fragmentation patterns reported in the literature: heterocyclic ring fission (m/z 411.07, m/z 125.02, m/z 449.09 and m/z 451.10), retro-Diels–Alder fission (m/z 407.07) and, most importantly, quinone methide cleavage (m/z 285.04, m/z 287.06, m/z 289.07, m/z 573.10 and m/z 575.12). Notably, fragment ion at m/z 289.07, corresponding to quinone methide cleavage of the interflavan bond, was detected for almost all compounds regardless of linkage type. Meanwhile, characteristic ions for A-type linkages at m/z 573.1 and m/z 285.04 were absent from fragmentation spectra of B-type PCs. The latter can only be produced when (epi)catechin is linked to an extension unit with an A-type linkage. Overall, full-MS and ddMS2 data complement each other, confirming that all detected peaks can be putatively identified as members of the PC family. More detailed information about each peak is available in the supplementary information (see Data S1 in the Supplementary Materials).

**Figure 6:**
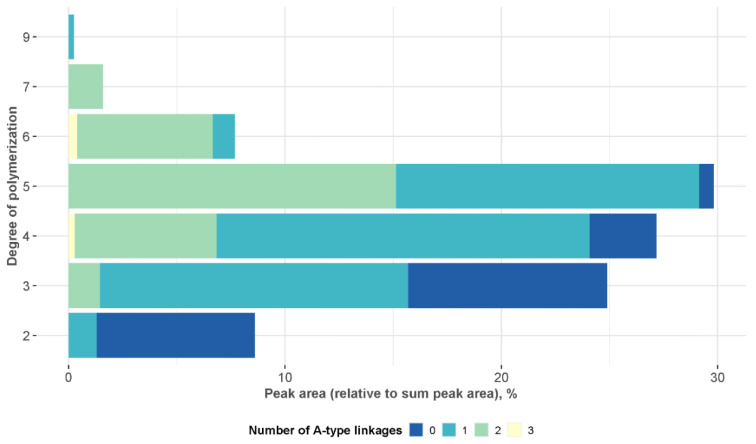
LC-HRMS peak area profiles of procyanidin oligomers found in *Vaccinium macrocarpon* extract, expressed as a percentage of the total peak area.

While the LC-HRMS method used in this study cannot yield quantitative data on individual constituents, relative peak area distribution can be used to estimate the PC oligomer composition profile. As seen in Figure 6, the largest peak areas were produced by PC pentamers (30%), tetramers (27%) and trimers (25%). Meanwhile, B-type dimers produced higher intensity chromatographic peaks compared to A-type dimers but, in general, A-type PCs were more dominant and accounted for almost 80% of the total peak area. Their prevalence increased along with the degree of polymerization (Figure 6).

## 3. Discussion

Cranberry phytochemicals have unique properties ranging from anti-cancer activity, cardiovascular disease prevention, anti-inflammatory effects and, most notably, UTI prevention. Despite the well-known UTI prevention ability and the fact that cranberry extracts containing procyanidins are being sold [25], their use in traditional medicine and the molecules—procyanidins—responsible for this activity, are not recognized due to the lack of clinical evidence. There have been several studies and clinical test to show the effects of procyanidins on UTI prevention; however, the studies all had limitations in respect of statistical feasibility and the lack of sufficient sample groups, thus the results are inconclusive. As the research methods have developed since the first trials on procyanidin uses for UTI prevention in early 1990s [26], the scientific interest of procyanidin research from cranberries and other types of material has switched to other health benefits of these phytochemicals. Cranberries are among the few foods that have the unique A-type procyanidins which are believed to have greater bioactivities than the B-type procyanidins; for this sole reason, the exploration of the chemical characteristics and bioactivity of cranberry procyanidins still remains an unexplored field which needs clarification.

Intervention studies are often conducted using milled cranberry powders or cranberry juice, which have relatively low concentrations of procyanidins. Extraction of cranberry procyanidins and the provision of pure fractions of these molecules could increase the effectiveness of such supplementation. Surveying previously published research articles on procyanidin extraction, it can be seen that there is no consensus on the extraction variables. The standardized extraction method performed in this study with the various extraction solvents reveals the need for standardization; the results vary depending on the extraction solvent used by several fold. Comparison of such data is difficult, since each study uses material with different origins and characteristics. In this study, it was shown that the best solvent to be used is acetone. In studies where acetone or methanol has been used as the solvent of choice, a variety of acidifying agents have also been added to the extraction medium [17]; for example, acetic acid [27,28,29], trifluoroacetic acid [30], formic acid [31] and hydrochloric acid [32]. While acidification of the extraction medium has been proven to aid polyphenolic extractions [33,34], it has not been demonstrated for the extraction of procyanidins [35].

Supporting the above-mentioned observation on acid influence for the increase of procyanidin extraction yields, the performed RSM optimization of solvent composition also shows that acidification of the procyanidin extraction medium does not contribute to the increase of the observed response. In an RSM study conducted by Diaz-de-Cerio et al., it can also be seen that the addition of acetic acid to acetone for extraction of procyanidins has an insignificant effect on the extraction yield [28]. The rationale behind the addition of acid can be attributed to the need for extraction of bound procyanidins. Procyanidins in cranberries or other types of materials can be divided in two general groups: free and conjugated (bound). Free procyanidins are usually the type of procyanidins which are analyzed; they can be extracted using solvents and the degree of polymerization is usually up to 10, while bound procyanidins are most commonly extracted using acid, alkaline or enzymatic hydrolysis [36]. Bound procyanidins are believed to have higher concentration in a specific material than the free; however, the activity is believed to be higher for the lower DP free procyanidins [37]. The concentration of acid in solvent assisted extractions is too low to ensure hydrolysis and the release of bound procyanidins; therefore, no changes can be seen with the addition of acid in the range 1–5%. Acid hydrolysis break down the glycosidic bond of the structural polysaccharides while enzymatic hydrolysis could be used to degrade cellulose, hemicellulose or pectin to release the bound procyanidins [37,38]. Such approaches, in combination with in-depth chromatographic studies, should be conducted to evaluate the actual total procyanidin contents of a specific material and the optimization of extraction should be conducted, preferably, by using RSM to evaluate the interactions between the extraction variables. This study, using RSM, showed that the optimal acetone concentration for the extraction of procyanidins from cranberry press residues is ≈53%. The most used acetone concentration for extraction of procyanidins is 70% for a variety of materials [39,40]. Using 70% acetone provides 30–35% less total procyanidin yields than the optimized conditions in this study. In the context of biorefining, the need for extraction methods is ever increasing; while the juice containing only free procyanidins is being produced and sold, the material that is left behind—the berry press residues—contain far higher concentrations of procyanidins that could be further refined to produce effective nutraceutical ingredients or products.

Purification and fractionation of the obtained crude cranberry press residue extract showed increasing antiradical scavenging activity with increasing procyanidin contents. The antioxidant activity of procyanidins is associated with the specific molecular stereostructure of the molecule and the presence of phenolic hydroxyl groups. Procyanidins have been shown as potential antioxidants in intervention studies; the positive effects have been compared to those of Vitamin E [41]. The structure and degree of polymerization are highly correlated to the antioxidant and free radical scavenging capacity of procyanidins. Procyanidins with higher DP are more effective when the oxidation is initiated by the lipid domains; these molecules are able to incorporate into the lipid membrane, thus providing protection from oxidative stress for hydrophobic and hydrophilic domains. On the other hand, monomers, dimers and trimers have been shown to be more effective when liposome oxidation happens. For a clearer understanding of the function of different type and DP procyanidins, they should be fractionated according to the bond type as well as DP. The bioavailability of larger procyanidins DP > 3 shows that these molecules have less contribution to physiological effects. Low concentration of procyanidins in the biomass was analyzed; their similarity and complexity limit the understanding of specific procyanidin molecules in the context of chemical characterization as well as in vitro or in vivo tests.

LC-ESI-FTICR-HRMS was used to determine the qualitative profile of the extracted and purified procyanidin fractions. In this study, 78 procyanidins were identified. The rapid development and availability of ultra-high accuracy HRMS allows for precise identification of complex matrices. The similarity of procyanidin molecules based on their monomer type, degree of polymerization and linkage type makes their identification tedious; moreover, the chromatographic separation of these compounds, due to their similarit, is difficult. Using advanced chromatographic and detection systems, these issues can be overcome; however, the analyzed fractions should still be considerably pure to avoid matrix effects that are introduced by the numerous interferences present in natural product extracts. For procyanidins of low concentration, FT-ICR is an excellent tool since it allows for the amplification of the signal to detectable levels. The increased sensitivity and resolution allow the successful identification of various phytochemicals; however, the complexity and costs of this type of analysis limits the application of this method. Another aspect of procyanidin analysis using HRMS methods is the lack of standards; the quantification, due to the number of specific molecular characteristics, is not currently possible. Orbitrap MS has been previously successfully used to identify a similar number of procyanidins in cranberries; however, these methods are limited to the maximum m/z that can be observed [42]. To further increase the number and DP of identified polymeric procyanidins, methods like TOF-MS should be used [43]. Use of FT-ICR with MALDI has been demonstrated for the elucidation of structural details of procyanidins, showing the effectiveness of these ultrahigh resolution methods [44].

The extraction, fractionation and analysis of procyanidins remain a challenge even with the most modern and precise methods. With procyanidins being a group of compounds with such complexity, standardized methods for extraction and analysis should be determined which will allow for more precise characterization of the biological activity.

## 4. Materials and Methods

### 4.1. Plant Material

American cranberry (*Vaccinium macrocarpon* L.) press residues were obtained from “Very Berry” Ltd. The obtained press residues have been flash-frozen at −20 °C immediately after the juice pressing. The total moisture of the press residues was 79%, pH 3.78 and 6.5 Brix. Upon delivery the press residues were placed into a drying oven at 40 °C and dried until no further change in mass was observed for 2 h. The drying of the press residues was conducted to avoid any unwanted dilution of the extraction solvents. Dried press residues were homogenized using a blade mill with sieve size 1 mm (IKA, Staufen, Germany) and stored at −20 °C until further use.

### 4.2. Extraction of Procyanidins

Extraction of procyanidins was conducted by weighing out 5.0 g of dried cranberry press residues into a 100 mL extraction vessel. We added 50 mL of the respective solvent mixture (acetone, methanol, water, ethanol, DMSO, 1.25 M NaCl solution together with trifluoroacetic acid, acetic acid, hydrochloric acid or formic acid, according to the experimental design) to the extraction vessel depending on the performed extraction: screening of solvent variables or optimization using RSM (according to the experimental design). The extraction vessel was placed in a 300 W (100 kHz) water-cooled ultrasound bath (Cole-Parmer, Chicago, IL, USA) for 15 min. After the ultrasonification, the extraction vessel was removed and the contents of the vessel were filtered through laboratory filter-paper. The filter paper was then placed back into the extraction vessel and another 50 mL of the respective solvent were added. The extraction process was repeated for a total of 3 times. The resulting extract filtrates were combined in 200 mL volumetric flasks and diluted to mark. The obtained extracts were stored at 4 °C until further analysis (within 24 h).

### 4.3. Determination of Group Parameters

#### 4.3.1. Determination of Total Anthocyanins

Quantification of total anthocyanins was conducted using a pH differential method [45] modified for a 96-well microplate reader (Tecan Nanoquant Infinite M200, Männedorf, Switzerland). Briefly, 0.025 M KCl buffer solution with pH 1.0 and 0.40 M sodium acetate buffer solution with pH 4.5 was prepared by adjusting the pH with conc. HCl. The sample was dissolved in an appropriate solvent (acetone) in the concentration 1 mg/mL. The sample was diluted with each of the buffer within the 96-well plate by taking 30 µL of the sample and 170 µL of each of the buffer. Samples were then incubated in shaking in the dark for 30 min. Samples were measured at 520 and 700 nm using dem. water as sample blank. The obtained absorbance values were used to calculate total anthocyanin contents in the sample using the following equation (Equation (1)):

(1) $$\mathrm{Total}\;\mathrm{anthocyanins}\;(\mathrm{cyanidin}\text{-}3\text{-}\mathrm{glucoside}\;\mathrm{eq}.,\;\mathrm{mg}/L)=\frac{A\cdot MW\cdot DF\cdot 10^{3}}{\varepsilon \cdot l}$$

where *A*—pH 1.0 (A520 nm–A700 nm) and pH 4.5 (A520 nm–A700 nm); *MW* (molecular weight) = 449.2 g/mol for cyanidin-3-glucoside; *DF* (dilution factor); *l*—pathlength in cm (x = cm); *ε*—26,900 molar extinction coefficient in L∙mol^−1^∙cm^−1^ for cyd-3-glu; 10^3^—factor for conversion from g to mg. Total anthocyanins have been expressed as ACN (total anthocyanins) mg or g/100 g of died cranberry press residues (*n* = 3).

#### 4.3.2. Determination of Total Polyphenolics

Quantification of total polyphenolics was conducted using the Folin–Ciocalteu method [46] modified for a 96-well microplate reader. Folin–Ciocalteu reagent (Sigma Aldrich, Hamburg, Germany) was diluted to 0.4 M. Anhydrous Na_2_CO_3_ was prepared in the concentration of 10% (*w/v*%) and a standard solution of gallic acid was prepared in the concentration of 1 mg/mL in 96% ethanol. All the wells were filled with 50 µL dem. water to which 50 µL of the sample or standard was added (diluted if necessary). Eighty-five milliliters of Folin–Ciocalteu reagent was added and incubated, shaking for 5 min. After, the incubation 65 µL Na_2_CO_3_ was added and incubated, shaking for 30 min. Measurements of absorbance of the samples and standards were taken after the incubation at 750 nm against a blank of reagents (using dem. water as sample). Calibration curve was then constructed, and the sample concentration was calculated using the regression equation. Total polyphenolics have been expressed as TPC (total polyphenolics) g/100 g of died cranberry press residues (*n* = 3).

#### 4.3.3. Determination of Total Procyanidins

Quantification of total procyanidins was conducted using 4-dimethylaminocinnamaldehyde (DMAC) colorimetric method [47] modified for a 96-well microplate reader. Procyanidin samples were dissolved in PAC extraction solvent (75% acetone, 24.5% water, 0.5% acetic acid *v/v/v*%). Acidified ethanol (75% ethanol, 12.5% water, 12.5% HCl *v/v/v*%) and DMAC reagent (0.05 g DMAC in acidified ethanol) were prepared. Standards were prepared using epicatechin in the concentration range 1–10 µg/mL and further used in the same manner as the samples. Standard measurements were used to construct a calibration curve and express a regression equation for the calculations of sample procyanidin contents. We added 100 µL of sample to 96-well plate and 200 µL of DMAC reagent was added. The sample plate was then placed into the plate reader and the absorbance was read every minute for 30 min at 640 nm using reagents with dem. water as sample blank. The highest absorbance within the 30-min measurement window was taken as sample absorbance and used for the calculations of total procyanidin (TPCA) concentration expressed as g/100 g dried cranberry press residues (*n* = 3).

### 4.4. Determination of Antioxidative Potential

#### 4.4.1. DPPH

A modified 2,2-Diphenyl-1-picrylhydrazyl (DPPH) antioxidant activity determination method was used to estimate radical scavenging potential [48]. DPPH stock solution was prepared by weighing 24 mg of DPPH and dissolving in 80% methanol. DPPH working solution was prepared by diluting 50 mL of the stock solution with 50 mL 80% methanol. Trolox was used as a standard in the concentration 1 mg/mL in ethanol. We added 50 µL of the respective sample or standard to the 96-well plate and 150 µL of the DPPH working solution was added. The plate was placed in the plate reader and pre-incubation period of 10 min was used to mix the sample (5 min orbital shaking at 2 mm amplitude). After, the incubation samples were measured at 517 nm. The concentration of Trolox equivalents was calculated according to the regression equation obtained and the results were expressed as g Trolox equivalents (TE)/100 g dried cranberry press residues (*n* = 5).

#### 4.4.2. FRAP

A modified ferric reducing antioxidant potential (FRAP) method was used to estimate the radical scavenging potential [49]. We prepared 0.010 M 2,4,6-Tripyridyl-s-triazine (TPTZ), 0.3 M sodium acetate solution at pH 3.6 and 0.02 M FeCl_3_∙6H_2_O solutions. The respective samples and standard (Trolox) were dissolved in ethanol in the concentration of 1 mg/mL. The FRAP working solution was prepared on the day of use by mixing sodium acetate buffer, TPTZ and FeCl_3_∙6H_2_O solution in the ratio of 10:1:1, respectively. We added 20 µL of sample or standard into the wells of the 96-well plate along with 180 µL of the FRAP reagent. The prepared samples were then incubated for 30 min after which the plate was read at 593 nm using ethanol and FRAP reagent as blank. The FRAP potential was calculated according to the constructed regression equation and expressed as TE g/100 g dried cranberry press residues (*n* = 5).

#### 4.4.3. ABTS

A modified 2,2′-azino-bis(3-ethylbenzothiazoline-6-sulfonic acid antioxidant) potential (ABTS) method was used to estimate the radical scavenging potential [49]. Phosphate buffer (PBS) was prepared by weighing 0.27 g of KH_2_PO_4_, 1.42 g Na_2_HPO_4_, 8.18 g NaCl and 0.15 g KCl in 1 L of dem. Water. pH was adjusted to 7.4 with NaOH. ABTS stock solution was prepared in the concentration of 2 mM by dissolving 0.10292 g of ABTS in 100 mL PBS buffer. Potassium persulfate (K_2_S_2_O_8_) solution in the concentration of 70 mM was prepared by weighing out 0.94613 g of K_2_S_2_O_8_ in 50 mL dem. water. Trolox was used as standard in the concentration 1 mg/mL. A day before the ABTS measurements the ABTS^+^ solution was prepared by reacting 50 mL ABTS stock solution with 200 µL K_2_S_2_O_8_ solution. The ABTS^+^ solution was then left in the dark for 16 h for the radical to develop. The next day ABTS^+^ solution was diluted to absorbance 0.800 with PBS buffer and used for the measurements. We mixed 50 µL of sample or standard (1–10 mg/mL) with ABTS^+^ solution and measured at 734 nm using PBS or sample solvent with ABTS^+^ as blank. The antioxidative potential was calculated using obtained regression equation and expressed as TE g/100 g dried cranberry press residues (*n* = 5).

### 4.5. Purification and Fractionation of Cranberry Press Residue Extract

A preparative-scale extraction of cranberry press residues was performed using the optimized extraction conditions (Section 2.2). We extracted 3.0 kg of dried cranberry press residues, which yielded 207.8 g of crude extract with the TPCA concentration 0.89 g/100 g press residues. The extract was concentrated using a rotary evaporator (Heidolph, Schwabach, Germany) and then dissolved to volume (1 L). The dissolved extract was loaded onto a column containing 500 g Amberlite XAD7-HP resin (pre-conditioned with dem-water, 5 CV). After loading, the column was washed with 3 CV dem. water to remove carbohydrates and soluble acids. Non-polar polyphenolic compounds were eluted using ethyl acetate (1 CV) and polar polyphenolics together with anthocyanins were eluted using ethanol acidified with formic acid (5%). Obtained ethyl acetate XAD fraction was then loaded onto Sephadex LH20 resin (pre-conditioned with 3 CV methanol). LH20 resin was first eluted using acidified ethanol, which allowed for the removal of co-pigments and any residual anthocyanins. LH20 resin was then eluted using methanol, which resulted in the removal of polyphenolic substances. LH20 resin was then eluted with 70% acetone, which yielded a procyanidin rich fraction. The procyanidin rich fraction was then loaded onto KP-SIL (silicagel) resin (pre-conditioned with 70% acetone). KP-SIL resin was eluted using an acetone gradient from 70% to 100% acetone. Four distinct bands formed within the column (monitored using UV) and were gathered separately yielding procyanidin fraction 1, 2, 3 and 4. The obtained fractions were then analyzed using LC-ESI-FTICR-MS. The purification process was repeated 3 times.

### 4.6. LC-ESI-FTICR-MS Analysis of Procyanidins

Liquid chromatography high resolution mass spectrometry (LC-HRMS) analysis of purified procyanidin extract was performed using a Dionex UltiMate 3000 UHPLC system (Thermo Fisher Scientific, San Jose, CA, USA). Chromatographic separation was carried out on a Phenomenex Luna C18(2) 100 A (100 × 2 mm, 3 μm) analytical column using a binary mobile-phase gradient, consisting of 0.1% formic acid in water (A) and acetonitrile (B). The gradient program was as follows: 2% (B) from 0 to 1 min, 2–10% (B) from 1 to 5 min, 10% (B) from 5 to 15 min, 10–20% (B) from 15 to 20 min, 20% (B) from 20 to 30 min, 20–90% (B) from 30 to 40 min, 90% (B) from 40 to 45 min and 2% (B) from 45 to 50 min. The flow rate was set to 0.25 mL/min and injection volume was 5 μL. Samples were repeatedly injected 5 times. Autosampler and column compartment temperatures were maintained at 14 °C and 40 °C, respectively.

The LC system was coupled to a 7T Bruker SolariX FT-ICR-HRMS system (Bruker Daltonics, Bremen, Germany) equipped with an electrospray ionization source (ESI). The ESI source was operated in negative ionization mode. Main ESI source parameters were set as follows: capillary voltage, 3 kV; nebulizer gas flow rate, 1.0 bar; drying gas temperature, 200.0 °C; drying gas flow rate, 8.0 L/min. Spectra were acquired over the range of m/z 50 to 2000. Accumulation time was set to 0.05 s. For full scan mode (full-MS) a transient size of 2 M was used (acquisition time of 0.73 s), which corresponds to a resolving power of around 66,000 FWHM (m/z 500). Data-dependent MS/MS (ddMS2) mode was operated using a transient size of 512 k (acquisition time 0.28 s), allowing the achievement of a resolving power of around 16,000 FWHM (m/z 500). The ddMS2 experiments were performed in three separate runs differing by fixed collision voltage value (10, 20 and 35 eV). Accurate mass calibration was performed once every five injections by introducing a 5 mM sodium formate solution via LC system using a 5 min isocratic mode which matched the initial mobile phase composition of the gradient program. Instrument control and data acquisition were performed with FTMS Control 2.2.0 software (Bruker Daltonics, Bremen, Germany). Data processing was carried out in Data Analysis 5.3 software (Bruker Daltonics, Bremen, Germany). The molecular formula of detected peaks was determined according to the accurate masses and isotopic pattern fit. The main parameters for molecular formula assignment in Data Analysis 5.3 software were as follows: electron configuration, even; permittable accurate mass error, ±2.5 ppm; maximum H/C ratio, 3; minimum H/C ratio, 0; elemental formula restrictions, CHNO.

### 4.7. Experimental Design and Statistical Analysis

Extraction variables were identified by performing extraction experiments using cranberry press residues with the respective solvent under the same extraction conditions (ultrasound assisted extraction) and range of the tested variables (acetone concentration, additive-acid concentration). The identified variables were further optimized using response surface methodology (RSM).

A two factor and three level central composite design (CCD) was performed (with three central points). Possible effects of unexplained variability due to extrinsic factors in the observed response (total procyanidins) were minimized by randomizing the run (experiment) order. The range and the levels of the variables chosen based on the preliminary results are summarized in Table 5.

The experimental design of CCD consisted of 52 experimental points with 6 central points (13 experiments with 4 repetitions for each tested solvent-acid combination). The data obtained for RSM was fitted to a second-order polynomial model with a generalized second-order (quadratic) model (Equation (2)):

(2) $$y=\beta_{0}+\overset{k}{\underset{j=1}{\sum }}\beta_{j}x_{j}+\overset{k}{\underset{j=1}{\sum }}\beta_{jj}x_{j}^{2}+\overset{\;}{\underset{i<j}{\sum }}\overset{k}{\underset{j=2}{\sum }}\beta_{ij}x_{i}x_{j}$$

where *β*_0_ is the regression coefficient for intercept, *β_j_* is the regression coefficient for linear terms, *β_jj_* is the regression coefficient for quadratic terms, *β_ij_* is the regression coefficient for interaction terms, and the independent variables are shown as *x_i_* and *x_j_*. Experimental data was first fitted to test the obtained model and the resulting prediction values were further used to generate a tri-dimensional regression model (surface plots). The RSM design and optimal extraction conditions were determined by maximizing desirability using a response profiler. All the statistical analysis and experimental design were created and tested using SAS JMP^®^ 17.0 software (Cary, NC, USA). The obtained fit statistics of the generated quadratic regression models were validated based on the determination coefficients by ANOVA and resulting *p*-values (a summary of fit statistics can be found in Table 6). ANOVA with post-hoc Tukey’s HSD was used to compare means between different treatments. Pairwise correlation was determined between the different variables tested. A paired *t*-test was used to determine statistical differences before and after the sample treatment.

## 5. Conclusions

Different extraction solvents commonly used for the extraction of procyanidins were compared to extract these substances from American cranberry press residues. The solvent providing the highest total procyanidin yield was acetone. In combination with an acidifying agent, the concentration of acetone was optimized using the response surface methodology (RSM). The optimal extract was prepared and purified to obtain procyanidin rich fractions, which were tested for their antioxidative properties and qualitative composition. Our study showed that the addition of acidifying agents for the extraction of procyanidins did not increase the procyanidin yields significantly over a gradient of acid and acetone concentrations. Additionally, with respect to the known procyanidin properties for the urinary tract infection prevention, it was shown that these phytochemicals have high radical scavenging properties. The use of a state-of-the-art chromatography method (LC-FT-ICR-HRMS) allowed the identification of 78 individual procyanidins with the degree of polymerization up to 9. Sixty-five of the identified procyanidins belonged to the A-type procyanidins, which is the conformation of procyanidins responsible for the specific E. coli anti-adhesion ability. Altogether, our results indicate the possible use of cranberry press residue in biorefinery strategies to obtain functional ingredients that could potentially benefit human health.

## Figures and Tables

**Table 1:** The effects tests of the predicted quadratic polynomial models for the investigated response—total procyanidins (α = 0.05).

Variable	Acetone-Acetic Acid			Acetone-Formic Acid			Acetone-Hydrochloric Acid		
	Sum of Sq.	*F* Ratio	*p*-Value	Sum of Sq.	*F* Ratio	*p*-Value	Sum of Sq.	*F* Ratio	*p*-Value
*X*_1_	1.029	219.24	<0.0001 *	2.781	1042.7	<0.0001 *	2.126	334.34	<0.0001 *
*X*_2_	<0.0001	<0.0001	0.9764	0.003	1.22	0.274	0.051	8.03	0.0068 *
*X*_1_ *X*_2_	<0.0001	0.0652	0.7995	0.004	1.58	0.214	0.002	0.39	0.5347

* represents a statistically significant effect.

**Table 2:** Optimized extraction conditions of total procyanidins from American cranberry press residues depending on extraction solvent composition.

Variable	Acetone-Acetic Acid	Acetone-Formic Acid	Acetone-Hydrochloric Acid
Acetone, % (*X*_1_)	53.3	52.8	53.7
Acid, % (*X*_2_)	2.30	0	2.50

**Table 3:** Quantitative analysis of group parameters in the purified fractions of cranberry press residue procyanidin extract. “Purification Step” represents the number of sample fractions during the purification process as indicated in Figure 3. RSD—relative standard deviation, *n* = 3.

	TPCA		TPC		ACN	
Purification Step	C, g/100 g	RSD, %	C, g/100 g	RSD, %	C, g/100 g	RSD, %
0	2.9	3.0	31.9	3.7	0.30	4.1
1	1.8	3.8	23.0	2.9	0.30	4.2
2	7.0	2.7	41.5	2.1	<LOD	
3	7.3	3.3	18.9	2.2	<LOD	
4	9.1	3.6	50.4	1.2	<LOD	
5	8.3	1.8	51.8	1.2	<LOD	
6	9.0	3.1	38.4	3.4	<LOD	
7	10	1.4	52.3	1.6	<LOD	
8	5.7	2.6	57.5	0.30	<LOD	

**Table 4:** Retention times, full-MS features and putative identities of the detected procyanidin oligomers in the purified *Vaccinium macrocarpon* procyanidin rich fraction. RT—retention time; (E)C—(epi)catechin; (E)GC—(epi)gallocatechin; DP—degree of polymerization.

No.	RT, min	m/z	Mass Error, ppm	Molecular Formula	Most Intense Species	Monomer	DP	Type (Number of A-Type Linkages)
1	9.84	879.1770	0.9	C_45_H_36_O_19_	[M-H]^−^	(E)C, (E)GC	3	A (1)
2	9.99	575.1195	−0.1	C_60_H_48_O_24_	[M-2H]^2−^	(E)C	4	A (1)
3	10.2	577.1349	0.5	C_30_H_26_O_12_	[M-H]^−^	(E)C	2	B
4	11.49	719.1508	0.6	C_75_H_60_O_30_	[M-2H]^2−^	(E)C	5	A (1)
5	11.55	576.1271	0.4	C_60_H_50_O_24_	[M-2H]^2−^	(E)C	4	B
6	11.58	865.1982	0.3	C_45_H_38_O_18_	[M-H]^−^	(E)C	3	B
7	11.89	879.1771	0.8	C_45_H_36_O_19_	[M-H]^−^	(E)C, (E)GC	3	A (1)
8	11.9	719.1508	0.6	C_75_H_60_O_30_	[M-2H]^2−^	(E)C	5	A (1)
9	12.25	719.1507	0.7	C_75_H_60_O_30_	[M-2H]^2−^	(E)C	5	A (1)
10	12.76	865.1988	−0.4	C_45_H_38_O_18_	[M-H]^−^	(E)C	3	B
11	12.77	577.1352	−0.1	C_30_H_26_O_12_	[M-H]^−^	(E)C	2	B
12	12.85	719.1512	0.1	C_75_H_60_O_30_	[M-2H]^2−^	(E)C	5	A (1)
13	13.32	719.1507	0.7	C_75_H_60_O_30_	[M-2H]^2−^	(E)C	5	A (1)
14	13.6	576.1271	0.4	C_60_H_50_O_24_	[M-2H]^2−^	(E)C	4	B
15	13.85	575.1193	0.3	C_60_H_48_O_24_	[M-2H]^2−^	(E)C	4	A (1)
16	14.03	576.1272	0.2	C_60_H_50_O_24_	[M-2H]^2−^	(E)C	4	B
17	14.33	863.1821	0.9	C_90_H_72_O_36_	[M-2H]^2−^	(E)C	6	A (1)
18	14.66	863.1817	1.4	C_45_H_36_O_18_	[M-H]^−^	(E)C	3	A (1)
19	14.78	862.1742	1.0	C_90_H_70_O_36_	[M-2H]^2−^	(E)C	6	A (2)
20	15.57	862.1747	0.4	C_90_H_70_O_36_	[M-2H]^2−^	(E)C	6	A (2)
21	15.95	575.1194	0.2	C_60_H_48_O_24_	[M-2H]^2−^	(E)C	4	A (1)
22	16.28	862.1744	0.8	C_90_H_70_O_36_	[M-2H]^2−^	(E)C	6	A (2)
23	16.54	863.1830	−0.1	C_45_H_36_O_18_	[M-H]^−^	(E)C	3	A (1)
24	17.3	865.1979	0.8	C_45_H_38_O_18_	[M-H]^−^	(E)C	3	B
25	18.05	719.1508	0.5	C_75_H_60_O_30_	[M-2H]^2−^	(E)C	5	A (1)
26	18.45	575.1194	0.2	C_60_H_48_O_24_	[M-2H]^2−^	(E)C	4	A (1)
27	19.56	575.1193	0.4	C_60_H_48_O_24_	[M-2H]^2−^	(E)C	4	A (1)
28	20.38	1006.206	0.5	C_105_H_82_O_42_	[M-2H]^2−^	(E)C	7	A (2)
29	20.89	720.1588	0.4	C_75_H_62_O_30_	[M-2H]^2−^	(E)C	5	B
30	21.2	865.1982	0.4	C_45_H_38_O_18_	[M-H]^−^	(E)C	3	B
31	21.21	719.1509	0.4	C_75_H_60_O_30_	[M-2H]^2−^	(E)C	5	A (1)
32	21.6	1006.206	0.5	C_105_H_82_O_42_	[M-2H]^2−^	(E)C	7	A (2)
33	21.65	719.1509	0.5	C_75_H_60_O_30_	[M-2H]^2−^	(E)C	5	A (1)
34	22.05	1295.278	−0.3	C_135_H_108_O_54_	[M-2H]^2−^	(E)C	9	A (1)
35	22.07	863.1834	−0.6	C_45_H_36_O_18_	[M-H]^−^	(E)C	3	A (1)
36	22.27	719.1514	−0.2	C_75_H_60_O_30_	[M-2H]^2−^	(E)C	5	A (1)
37	22.29	576.1277	−0.7	C_60_H_50_O_24_	[M-2H]^2−^	(E)C	4	B
38	22.47	1006.207	−0.3	C_105_H_82_O_42_	[M-2H]^2−^	(E)C	7	A (2)
39	22.54	862.1749	0.2	C_90_H_70_O_36_	[M-2H]^2−^	(E)C	6	A (2)
40	22.66	575.1197	−0.4	C_60_H_48_O_24_	[M-2H]^2−^	(E)C	4	A (1)
41	22.89	573.1040	−0.3	C_60_H_44_O_24_	[M-2H]^2−^	(E)C	4	A (3)
42	23.02	863.1828	1.4	C_90_H_72_O_36_	[M-2H]^2−^	(E)C	6	A (1)
43	23.17	575.1198	−0.6	C_60_H_48_O_24_	[M-2H]^2−^	(E)C	4	A (1)
44	23.4	718.1440	−0.8	C_75_H_58_O_30_	[M-2H]^2−^	(E)C	5	A (2)
45	23.6	574.1120	−0.5	C_60_H_46_O_24_	[M-2H]^2−^	(E)C	4	A (2)
46	23.71	719.1516	−0.5	C_75_H_60_O_30_	[M-2H]^2−^	(E)C	5	A (1)
47	23.8	575.1199	−0.6	C_60_H_48_O_24_	[M-2H]^2−^	(E)C	4	A (1)
48	23.93	719.1514	−0.3	C_75_H_60_O_30_	[M-2H]^2−^	(E)C	5	A (1)
49	24.07	718.1445	−1.5	C_75_H_58_O_30_	[M-2H]^2−^	(E)C	5	A (2)
50	24.15	577.1359	−1.2	C_30_H_26_O_12_	[M-H]^−^	(E)C	2	B
51	24.23	575.1200	−0.9	C_30_H_24_O_12_	[M-H]^−^	(E)C	2	A (1)
52	24.32	862.1756	−0.7	C_90_H_70_O_36_	[M-2H]^2−^	(E)C	6	A (2)
53	24.33	719.1519	−1.0	C_75_H_60_O_30_	[M-2H]^2−^	(E)C	5	A (1)
54	24.48	863.1847	−2.1	C_45_H_36_O_18_	[M-H]^−^	(E)C	3	A (1)
55	24.59	575.1202	−1.1	C_60_H_48_O_24_	[M-2H]^2−^	(E)C	4	A (1)
56	24.77	718.1438	−0.6	C_75_H_58_O_30_	[M-2H]^2−^	(E)C	5	A (2)
57	24.84	865.1994	−1.0	C_45_H_38_O_18_	[M-H]^−^	(E)C	3	B
58	24.93	719.1516	−0.5	C_75_H_60_O_30_	[M-2H]^2−^	(E)C	5	A (1)
59	25.08	575.1204	−1.6	C_60_H_48_O_24_	[M-2H]^2−^	(E)C	4	A (1)
60	25.15	863.1846	−2.0	C_45_H_38_O_18_	[M-H]^−^	(E)C	3	B
61	25.44	862.1764	−1.6	C_90_H_70_O_36_	[M-2H]^2−^	(E)C	6	A (2)
62	25.44	719.1521	−1.2	C_75_H_60_O_30_	[M-2H]^2−^	(E)C	5	A (1)
63	25.62	574.1128	−1.9	C_60_H_46_O_24_	[M-2H]^2−^	(E)C	4	A (2)
64	25.66	718.1449	−2.1	C_75_H_58_O_30_	[M-2H]^2−^	(E)C	5	A (2)
65	25.78	575.1205	−1.8	C_60_H_48_O_24_	[M-2H]^2−^	(E)C	4	A (1)
66	25.97	718.1444	−1.5	C_75_H_58_O_30_	[M-2H]^2−^	(E)C	5	A (2)
67	26.13	861.1685	1.3	C_45_H_34_O_18_	[M-H]^−^	(E)C	3	A (2)
68	26.43	719.1514	−0.3	C_75_H_60_O_30_	[M-2H]^2−^	(E)C	5	A (1)
69	26.86	862.1751	−0.1	C_90_H_70_O_36_	[M-2H]^2−^	(E)C	6	A (2)
70	27.24	574.1126	−1.7	C_60_H_46_O_24_	[M-2H]^2−^	(E)C	4	A (2)
71	27.52	718.1436	−0.3	C_75_H_58_O_30_	[M-2H]^2−^	(E)C	5	A (2)
72	27.91	718.1439	−0.7	C_75_H_58_O_30_	[M-2H]^2−^	(E)C	5	A (2)
73	28.14	862.1750	0.1	C_90_H_70_O_36_	[M-2H]^2−^	(E)C	6	A (2)
74	28.36	574.1118	−0.2	C_60_H_46_O_24_	[M-2H]^2−^	(E)C	4	A (2)
75	29.39	863.1826	0.3	C_45_H_36_O_18_	[M-H]^−^	(E)C	3	A (1)
76	29.69	861.1670	0.3	C_45_H_34_O_18_	[M-H]^−^	(E)C	3	A (2)
77	31.85	861.1669	0.4	C_90_H_68_O_36_	[M-2H]^2−^	(E)C	6	A (3)
78	33.25	861.1666	0.8	C_45_H_34_O_18_	[M-H]^−^	(E)C	3	A (2)

**Table 5:** Independent variables and their levels used in the response surface design.

Independent Variables	Symbol	Coded Factor Levels		
		−1	0	+1
Acetone, %	*X*_1_	0	30	100
Added acid, % *	*X*_2_	0	2.5	5

* FA—formic acid; AA—acetic acid; HCI—hydrochloric acid.

**Table 6:** Analysis of variance (ANOVA) for the response surface models on the total procyanidin yield from American cranberry press residues.

**Sources**	**Degrees of Freedom**	**Sum of Squares**	**Mean Square**	**Coefficient**	***F*-Value**	***p*-Value**
**Response: Total Procyanidins (Acetone-Acetic Acid)**						
Model	5	2.617	0.523		111.50	<0.0001
Lack of fit	3	0.036	0.012		35.22	0.0469
Pure error	43	0.179	0.004			
Error	46	0.215	0.011			
Cor. total	51	2.832				
Cor. total R^2^	52			0.9237		
Adj—R^2^				0.9155		
**Response: Total Procyanidins (Acetone-Formic Acid)**						
Model	5	3.70	0.74		277.75	<0.0001
Lack of fit	3	0.0177	0.005		5.22	<0.0001
Pure error	43	0.136	0.002			
Error	46	0.122	0.122			
Cor. total	51	3.82				
Cor. total R^2^	52			0.9679		
Adj—R^2^				0.9644		
**Response: Total Procyanidins (Acetone-Hydrochloric Acid)**						
Model	5	6.15	1.23		192.573	<0.0001
Lack of fit	3	0.086	0.028		5.988	0.0017
Pure error	43	0.207	0.004			
Error	46	0.293	0.006			
Cor. total	51	6.450				
Cor. total R^2^	52			0.9544		
Adj—R^2^				0.9495		

## Supplementary Materials

The following supporting information can be downloaded at: https://www.mdpi.com/article/10.3390/plants11243517/s1, Figure S1: The top ten most frequently detected fragments across the ddMS2 dataset at a collision energy of 20 eV; Data S1: Full HRMS data of the identified procyanidins.
