# Supplementary material for: Procyanidins from Cranberry Press Residues—Extraction Optimization, Purification and Characterization
Source: Plants (Basel). 2022 Dec 14;11(24):3517. doi: 10.3390/plants11243517 (PMC9786595; doi:10.3390/plants11243517)

Figure S1. The top ten most frequently detected fragments across the ddMS^2 dataset at a collision energy of 20 eV

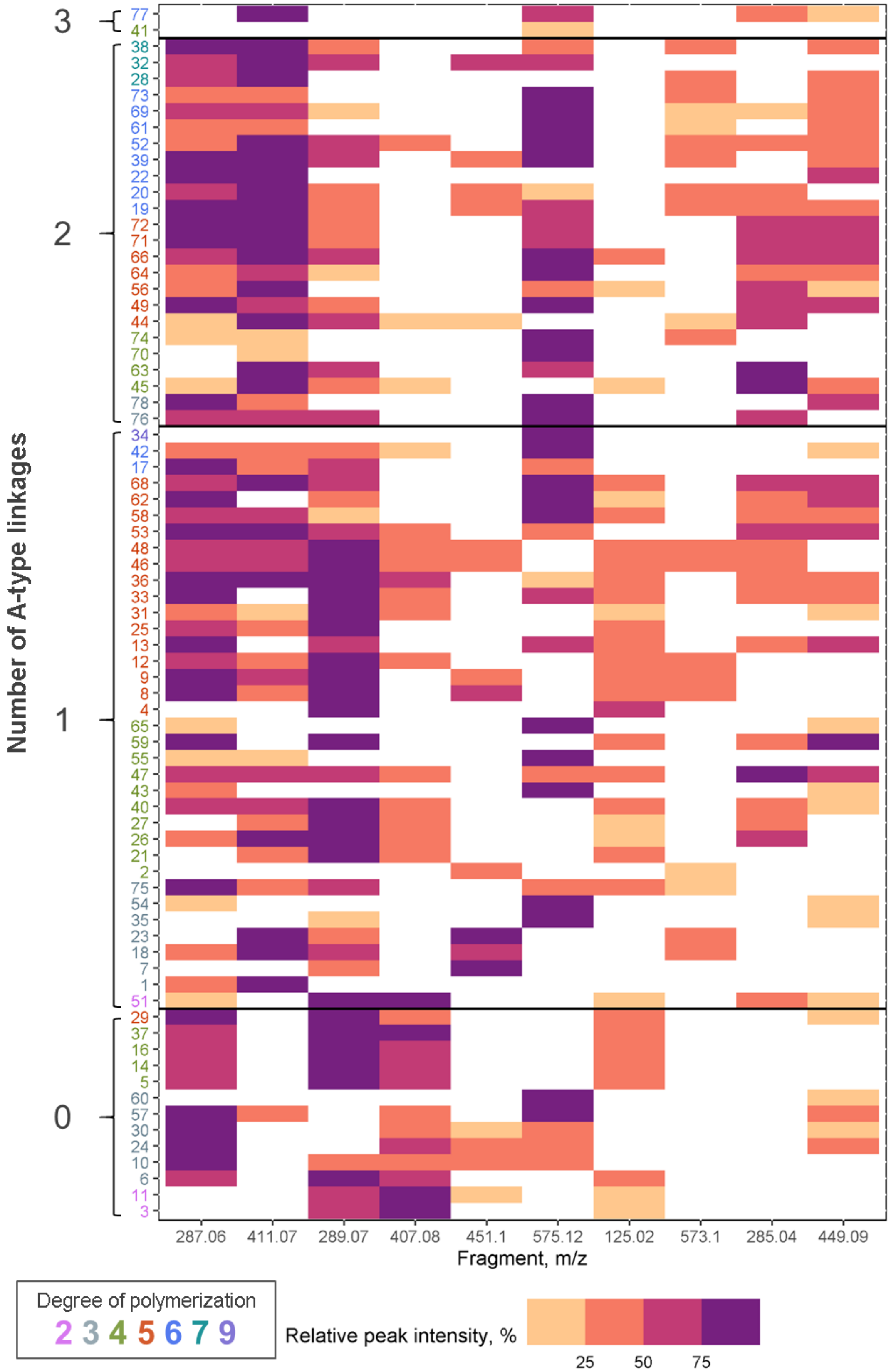

Supplement: Supplementary file 1 [file plants-11-03517-s001.zip › Figure S1.pdf]
